# Clindamycin Efficacy in Patients With Methicillin-Sensitive Staphylococcus aureus in a Fourth-Level Hospital in the City of Medellín

**DOI:** 10.7759/cureus.21124

**Published:** 2022-01-11

**Authors:** Jose C Alvarez-Payares, Jair E. Palacios M, Edwin De la Peña, Huxdley B. Cabrera, Santiago Giraldo-Ramírez, Marcela Loaiza, Fabian Jaimes, Joaquin Rodelo, Jose Ágamez-Gómez

**Affiliations:** 1 Departamento de Medicina Interna, Universidad de Antioquia, Medellín, COL; 2 Departamento de Medicina Interna, Hospital Universitario San Vicente Fundación, Medellín, COL; 3 Facultad de Medicina, Universidad de Antioquia, Medellín, COL

**Keywords:** treatment choices, bloodstream infections, clindamycin, mssa bacteremia, methicillin-sensitive staphylococcus aureus

## Abstract

Background: The antibiotic of choice for methicillin-sensitive *Staphylococcus aureus* (MSSA) bacteremia is antistaphylococcal penicillins, such as oxacillin, but cefazolin has also risen as an equally effective alternative. Murine models have suggested that clindamycin is a therapeutic alternative for *Staphylococcus aureus* bacteremia (SAB).

Methods: In this retrospective cohort study, patients from the Hospital Universitario San Vicente Fundación (HUSVF) in Medellín, Colombia, were recruited from January 2013 and December 2019. Patients with positive blood culture for MSSA, with at least one follow-up blood culture, and those with more than 72 hours of parenteral antibiotic therapy for SAB were selected. The main objective was to determine the efficacy of clindamycin compared to other antibiotics to achieve a microbiologic cure. Secondary results included in-hospital mortality and hospital stay.

Results: A total of 486 patients were included (clindamycin = 50 and other anti-MSSA = 436). The patients in the clindamycin group had a lower rate of microbiological cure (n = 41 [84%]) compared to other antibiotics (n = 367 [84%]) (OR 1.08 IC 95% 0.74-1.58). In secondary outcomes, no statistically significant differences were observed in the in-hospital mortality. The main source of SAB was a central or peripheral catheter (58%).

Conclusions: Our study found no differences in the rate of microbiological cure, in-hospital mortality, and hospital stay on the clindamycin group compared to other anti-MSSA antibiotics. However, in patients with metastatic complications, the rate of microbiological cure is reduced, and the in-hospital mortality is higher in patients with more severe disease.

## Introduction

*Staphylococcus aureus* (SA) is one of the most frequent causes of bacteremia in the hospitals of the United States [1,2]. It is a public health concern, associated with a mortality rate of 20%-30% in adults and 5% in children [3]. Historically, antibiotics of choice for methicillin-sensitive *Staphylococcus aureus* (MSSA) have been semisynthetic penicillins. Nonetheless, the treatment is complex due to the virulence, antibiotic resistance, high frequency of therapeutic failure, and scarce alternative treatment [2]. This has led to the search for new management options.

In toxin-mediated SA infections, such as necrotizing fasciitis, some guidelines recommend adding clindamycin to the standard treatment. These recommendations are based on expert opinions with limited clinical evidence [4,5]. Animal studies and human observational studies suggest the benefit from clindamycin [6,7]; however, no clinical trials sustain this strategy in SA bacteremia (SAB).

Concern surrounds the clindamycin’s efficacy, specifically in a bacteremia scenario - whether it is related to its bacteriostatic effect or not. Furthermore, clinical evidence supporting its use in this context is scarce [3]. Hence, the objective of the present study was to determine the efficacy of clindamycin treatment compared with other antibiotics in patients with SAB diagnosis in a fourth-level hospital.

## Materials and methods

Study design and population

This is a historical cohort study. Patients who were cared for at the Hospital Universitario San Vicente Fundación (HUSVF) in Medellín, Colombia, from January 2013 to December 2019 were recruited. The study was approved by the hospitals’ Ethics Committee and Investigation Directorate, and the approval number was 24-2019.

Patients

Patients who were 18 years or older with a positive MSSA blood culture, at least one follow-up blood culture, and at least 72 hours of parenteral antibiotic therapy were included. Patients with SAB diagnosis in the 30 days before hospitalization, combined antibiotic therapy or polymicrobial cultures, were excluded.

Variables

Patients were defined as exposed to clindamycin when parenteral antibiotic treatment was administered for at least 72 hours. Non-exposed patients were defined as those who received other anti-MSSA antibiotics (oxacillin, ceftriaxone, cefazolin, piperacillin/tazobactam, and ampicillin/sulbactam).

The primary outcome was a microbiological cure, which is defined as the absence of bacteria in control blood culture after 72 hours of anti-MSSA-directed therapy [8]. Secondary outcomes were as follows: (1) Hospital stay measured as days after the first positive blood culture up to discharge in survivors and (2) in-hospital mortality.

Confounding variables were selected based on the available literature, such as age, severity based on Sequential Organ Failure Assessment Score (SOFA) [9]. In patients for whom information was not available, severity was assessed according to ICU admission, immunosuppression (immunocompromise is defined as neutropenia ≤ 500 cells/mm^3^, systemic steroid use for more than a month, transplant patients, biologic drug use, or cancer chemotherapy), lactate > 2 mmol/L, and metastatic complications [8,10,11].

Data source

Patients were drawn from MSSA positive blood cultures from the time frame previously described. The electronic health record was reviewed to evaluate the demographical and clinical characteristics as well as the antibiotic treatment. Data were collected from January 2020 to February 2021.

Bias control

An information bias was identified, and it was mitigated through the definition of minimum variables of every patient to be included in the database, during the study period.

Sample size

No formal sample size calculation was performed as the entire population available in the cohort was analyzed.

Statistical analysis

Microbiological cure and in-hospital mortality were compared between groups (patients treated with clindamycin and patients treated with other antibiotics) through the chi-square test of independence or Fisher’s exact test according to the expected value in the cell. In-patient stance days were compared through the Mann-Whitney U test. For an adjusted analysis of microbiological cure and in-hospital mortality, according to the confounding variables previously defined, multivariable logistic analyses were performed with previous validation of the assumptions of the absence of multicollinearity, absence of interaction, and absence of linearity among continuous independent variables and the respective outcome logit. Results are shown as odds ratio (OR) with a 95% confidence interval (CI).

## Results

Patients

**Figure 1:**
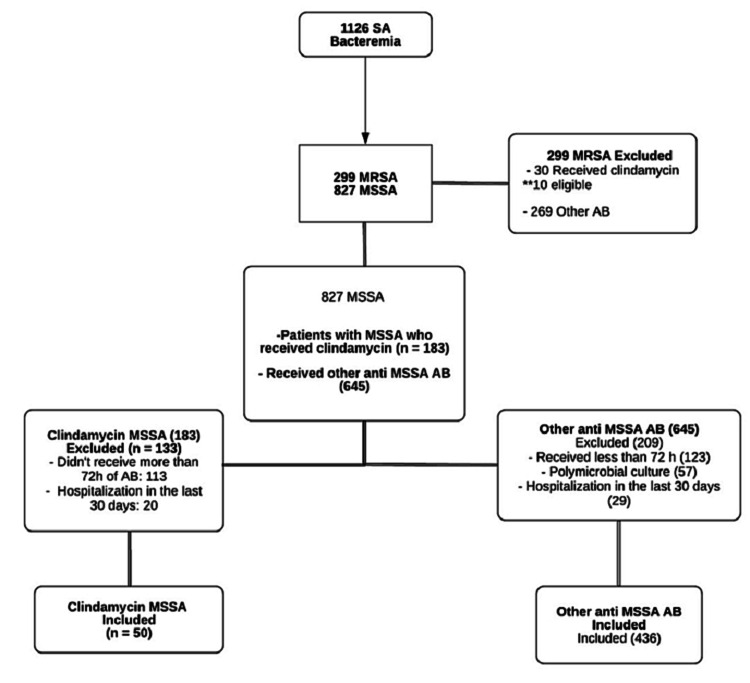
Recruitment, assignation, and inclusion in the primary analysis SA, *Staphylococcus aureus*; MRSA, methicillin-resistant *Staphylococcus aureus*; MSSA, methicillin-sensitive *Staphylococcus aureus*.

A total of 1126 charts from patients with SAB (including MSSA and MRSA) were reviewed, from a fourth-level hospital in Medellín, in the time-lapse of January 2013 and December 2018. After inclusion and exclusion criteria were applied, 486 patients with MSSA bacteremia were obtained, with 50 in the clindamycin group and 436 in the other antibiotics group (Figure 1). The information was collected from January 2020 to February 2021.

Patients’ characteristics

The mean age was 53.5 years (±17.9), predominantly male (58%) and mestizo (87%). The most frequent comorbidities were advanced chronic kidney disease (49%), diabetes mellitus (27%), and cancer (10%). The main source of SAB was a central or peripheral catheter (58%). One-third of the patients had lactate levels > 2 mmol/L, and a fifth required admission to the intensive care unit. In the group of other antibiotics with MSSA coverage, 50% received ceftriaxone, and 26.5% received cefazolin; most of the time, patients received empirical therapy (82%), mainly with vancomycin. The patients in the clindamycin group (88.25%) received an IV dose of 900 mg TID. The most common metastatic complications were pulmonary (21.57%) and osteomuscular ones (17.91%) (Table 1).

**Table 1:** Patient’s characteristics CV, Cardiovascular; CNS, central nervous system; CHF, congestive heart failure; CKD, chronic kidney disease defined as an eGFR < 30 mL/min; eGFR, estimated glomerular filtration rate.

Variables	Clindamycin, N = 50 (10.28%)	No clindamycin, N = 436 (89.71%)	p values
Demographical			
Age, Mean ± SD	56.5 ± 17.4	52.6 ± 18.1	0.052
Sex n (%), Male	31 (62%)	254 (58%)	0.446
Comorbidities			
Immunosuppression, any cause n (%)			
Neutropenia < 500	1 (2%)	8 (2%)	<0.001
Systemic steroid use for more than a month	4 (8%)	52 (12%)	<0.001
Transplant	0 (0%)	13 (3%)	<0.001
Biologic drugs	1 (2%)	20 (5%)	0.015
Cirrhosis	5 (10%)	14 (3%)	0.003
Cancer	5 (10%)	35 (8%)	0.015
HIV with CD4 < 200	3 (6%)	3 (0.7%)	0.002
Advanced CKD	16 (32%)	227 (52%)	0.019
CV disease			
CHF	17 (34%)	54 (12%)	0.019
Diabetes mellitus	11 (22%)	115 (26%)	0.549
Primary source of SA bacteremia			
Unknown	5 (10%)	42 (10%)	<0.001
Central/peripheral catheter	32 (64%)	240 (55%)	<0.001
Abscess	2 (4%)	28 (6%)	0.666
Cellulitis	7 (14%)	10 (2%)	<0.001
SOFA score	1 (0–2)	4 (1–6)	<0.001
Lactate > 2 mmol/L	10 (23%)	142 (34%)	0.039
Vasopressor/inotrope use	4 (8%)	49 (11%)	0.109
Ventilatory support	5 (10%)	49 (11%)	0.243
ICU requirement	6 (12%)	96 (22%)	0.211
Empirical antibiotic	41 (82%)	346 (79%)	0.003
Empirical treatment duration (days)	3 (2–3)	3 (2–3)	0.449
Directed therapy duration (days)	14 (10–16)	14 (9–17)	0.002
Metastasis complications			
Endocarditis	0	34 (8%)	0.010
Pulmonary			
Pneumonia	9 (18%)	70 (16%)	<0.001
Osteomuscular			
Osteomyelitis	10 (20%)	58 (13%)	<0.001
Septic arthritis	0	8 (2%)	<0.001
Skin	4 (4%)	31 (7.11%)	
Cellulitis	3 (6%)	17 (4%)	0.010
Abscesses	0	28 (6.4%)	0.160
CNS	1 (0.9%)	13 (3%)	

Main results

**Figure 2:**
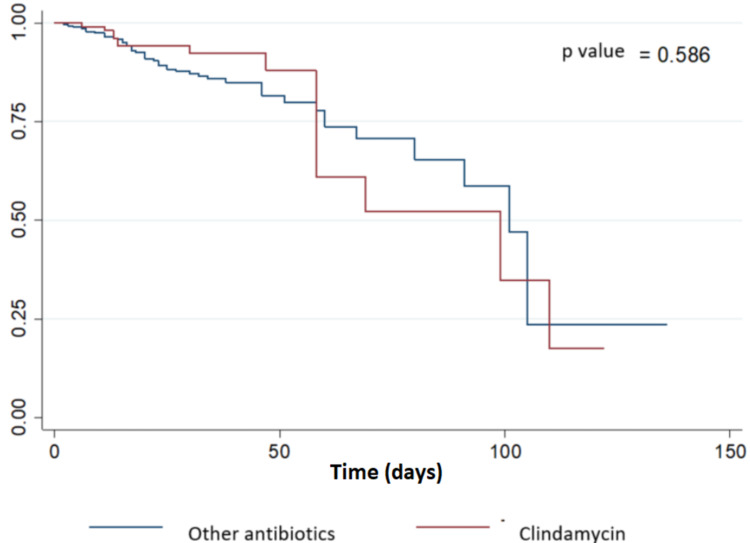
Survival curve of patients with MSSA bacteremia treated with clindamycin or other antibiotics MSSA, Methicillin-sensitive *Staphylococcus aureus*.

The group of patients treated with clindamycin for SAB achieved a microbiological cure in 82% of cases (n = 41) compared to 84% (n = 367) in the group of other antibiotics (OR 1.08 IC 95% 0.74-1.58). In secondary outcomes, no statistically significant differences in the in-hospital mortality were found (Figure 2); however, a significant difference in the metastatic complications was observed (20% vs 26%). All the clinical outcomes are highlighted in Table 2.

**Table 2:** Primary and secondary outcomes

Outcome	Clindamycin, N = 50 (10.28%)	No clindamycin, N = 436 (89.71%)	p value
Primary			
Microbiological cure	41 (82%)	367 (84%)	<0.001
Secondary			
In-hospital mortality	15 (14%)	56 (13%)	0.851
Stay hospital (days)	28 (17–42)	21 (15–36)	0.011
Persistant bacteremia	10 (20%)	113 (26%)	0.1
Microbiological clearance time (days)	3 (3-4)	3 (3-5)	0.078
Metastatic complications	13 (26%)	142 (33%)	<0.001

Multivariate analysis

After the multivariate analysis was performed, with the confounding variables previously established in the protocol, an OR of 1.45 (CI 95% 0.88-2.39) for the microbiological cure and 0.74 (CI 95% 0.36-1.54) for in-hospital mortality were found (Tables 3, 4).

**Table 3:** Association between MSSA bacteremia treated with clindamycin or other antibiotics with microbiological cure after multivariate analysis Immunocompromise is defined as neutropenia ≤500 cells/mm^3^, systemic steroid use for more than a month, transplant patients, biologic drug use, or cancer chemotherapy. MSSA, Methicillin-sensitive *Staphylococcus aureus*; SOFA, Sequential Organ Failure Assessment Score.

Variables	OR	CI 95%	Adjusted OR	Adjusted CI 95%
Anti-MSSA antibiotics other than clindamycin	1.08	0.74–1.58	1.45	0.88–2.39
Age	0.99	0.97–1.00	0.99	0.98–1.00
Immunocompromise	1.40	0.76–2.57	1.90	0.90–4.01
Lactate > 2 mmol/L	0.26	0.16–0.42	0.55	0.29–1.04
ICU admission	0.22	0.13–0.37	0.63	0.31–1.29
SOFA score	0.78	0.73–0.84	0.85	0.76–0.94
Metastatic complications	0.22	0.13–0.36	0.27	0.15–0.51

**Table 4:** Association between MSSA bacteremia treated with clindamycin or other antibiotics with in-hospital mortality after multivariate analysis Immunocompromise is defined as neutropenia ≤500 cells/mm^3^, systemic steroid use for more than a month, transplant patients, biologic drug use, or cancer chemotherapy. MSSA, Methicillin-sensitive *Staphylococcus aureus*; SOFA, Sequential Organ Failure Assessment Score.

Variables	OR	CI 95%	Adjusted OR	Adjusted CI 95%
Anti-MSSA antibiotics other than clindamycin	1.15	0.71–1.87	0.74	0.36–1.54
Age	1.03	1.01–1.04	1.04	1.01–1.06
Immunocompromise	1.38	0.76–2.50	2.12	0.95–4.76
Lactate > 2 mmol/L	9.35	5.01–17.43	3.92	1.61–9.55
ICU admission	9.74	5.41–17.55	3.47	1.58–7.62
SOFA score	1.48	1.35–1.63	1.26	1.13–1.41
Metastatic complications	2.92	1.69–5.05	1.96	0.92–4.21

## Discussion

Adequate antibiotic therapy is fundamental to prevent adverse outcomes associated with SAB [1]; therefore, the selection of an antimicrobial agent is a critical step. In our institution, the use of clindamycin as monotherapy in SAB has been driven by local studies in murine models that support its use in severe infections. In a neutropenic murine model, it was observed that clindamycin had bactericidal activity and the capacity to achieve a drop of more than three logs of colony-forming units (CFU), which equates to a bacterial inoculum > 99.9%, indicating a potent in vivo bactericidal effect [7].

We believe that this is the biggest cohort published until this date evaluating clindamycin’s role for SAB treatment. In this retrospective cohort, we found no statistically significant difference favoring in relation to microbiological cure, even when confounding factors were adjusted. As no similar study testing clindamycin’s efficacy in SAB treatment in humans has been reported, our results cannot be contrasted with other clinical studies. Nevertheless, the microbiological cure rate with clindamycin differs from those reported In the literature for ceftriaxone, cefazolin, and nafcillin/oxacillin (93.1%, 87.2%, and 91.2%, respectively) [12,13].

On multivariate analysis, we found that the primary outcome was reduced on patients in the clindamycin group with higher SOFA scores and metastatic complications. This suggests that clindamycin may not be a good alternative in patients with severe infections and complicated bacteremia.

Stay hospital and in-hospital mortality were similar in both groups; even after confounding factors were analyzed, no differences were observed (OR 0.74, IC 95% 0.36-1.54). There are some factors related to this outcome, such as one reported by a prospective multicentric European study of 987 patients, in which the persistence of SAB, defined in different times, was independently associated with mortality (≥2 days, HR 1.93; IC 95% 1.51-2·46) [8]. In our study, no significant difference in the microbiological clearance time was observed, which was higher than three days in both groups; nonetheless, one of the limitations of the present study was that the first control blood culture was taken at 72 hours from the first one.

Additionally, factors such as age, blood lactate > 2 mmol/L, ICU admission, and SOFA score were associated with increased mortality as was previously described in other studies [9,14-17]. Immunocompromised state and metastatic complications were not associated with higher mortality. The previous retrospective analysis did not identify immunocompromise as a mortality risk factor in SAB [18], although some studies did show an association [19-20]. Another important aspect is that it is known that endocarditis and pulmonary infection sources increase mortality, but no representative sample was found in neither group [18].

This study has some limitations such as its retrospective nature, conditioning a risk for incomplete information, the incapacity to determine all the confounding factors that may have a potential impact on treatment failure, e.g., control blood cultures were not taken daily, which can lead to uncertainty about the real duration of bacteremia. This has a direct impact on mortality rates [8]. Despite these limitations, this is a rare study comparing clindamycin with other antibiotics in MSSA bacteremia. Further studies are required to determine if clindamycin is equally effective as other anti-MSSA antibiotics in SAB treatment regarding microbiological cure, metastatic complications, and death. Even though the standard treatment for MSSA bacteremia is antistaphylococcal beta-lactams such as oxacillin or cefazolin, evidence is deficient and is based on observational studies [2].

## Conclusions

This is a rare study to explore the use of clindamycin for SAB. Our results suggest that different antistaphylococcal antibiotics for SAB treatment regarding microbiologic cure and intra-hospital mortality may be an option. However, in patients with metastatic complications, microbiological cure in the clindamycin group was inferior compared to other antibiotics. The differences in comorbidities, the type of study, and the patient's sample may explain the results; hence, further studies, ideally prospective ones, are required to evaluate the clindamycin’s role in this scenario.
